# The NEDD8 modification pathway in plants

**DOI:** 10.3389/fpls.2014.00103

**Published:** 2014-03-21

**Authors:** Julia Mergner, Claus Schwechheimer

**Affiliations:** Plant Systems Biology, Technische Universität MünchenFreising, Germany

**Keywords:** CAND1, COP9 signalosome (CSN), cullin, E3 ubiquitin ligase, F-BOX PROTEIN (FBP), NEDD8, RELATED TO UBIQUITIN (RUB), ubiquitin

## Abstract

NEDD8, in plants and yeasts also known as RELATED TO UBIQUITIN (RUB), is an evolutionarily conserved 76 amino acid protein highly related to ubiquitin. Like ubiquitin, NEDD8 can be conjugated to and deconjugated from target proteins, but unlike ubiquitin, NEDD8 has not been reported to form chains similar to the different polymeric ubiquitin chains that have a role in a diverse set of cellular processes. NEDD8-modification is best known as a post-translational modification of the cullin subunits of cullin-RING E3 ubiquitin ligases. In this context, structural analyses have revealed that neddylation induces a conformation change of the cullin that brings the ubiquitylation substrates into proximity of the interacting E2 conjugating enzyme. In turn, NEDD8 deconjugation destabilizes the cullin RING ligase complex allowing for the exchange of substrate recognition subunits via the exchange factor CAND1. In plants, components of the neddylation and deneddylation pathway were identified based on mutants with defects in auxin and light responses and the characterization of these mutants has been instrumental for the elucidation of the neddylation pathway. More recently, there has been evidence from animal and plant systems that NEDD8 conjugation may also regulate the behavior or fate of non-cullin substrates in a number of ways. Here, the current knowledge on NEDD8 processing, conjugation and deconjugation is presented, where applicable, in the context of specific signaling pathways from plants.

## NEDD8 IS AN EVOLUTIONARILY CONSERVED REGULATOR

NEDD8 (neural precursor cell expressed, developmentally down-regulated8), in plants and yeasts also known as RELATED TO UBIQUITIN (RUB, hitherto referred to as NEDD8), is a 76 amino acid protein that was originally identified as a highly expressed gene from embryonic mouse brains (Kumar et al., 1993). Amongst all ubiquitin-like modifiers (UBLs), NEDD8 and ubiquitin are most closely related to each other and NEDD8 proteins, like other UBLs, display remarkable sequence conservation across species (Vierstra, 2012). Like ubiquitin, NEDD8 is conjugated to its substrate protein through the formation of an isopeptide bond between its C-terminal glycine and a lysine residue of the target protein (neddylation) but there is no known biological function for free NEDD8.

**FIGURE 1:**
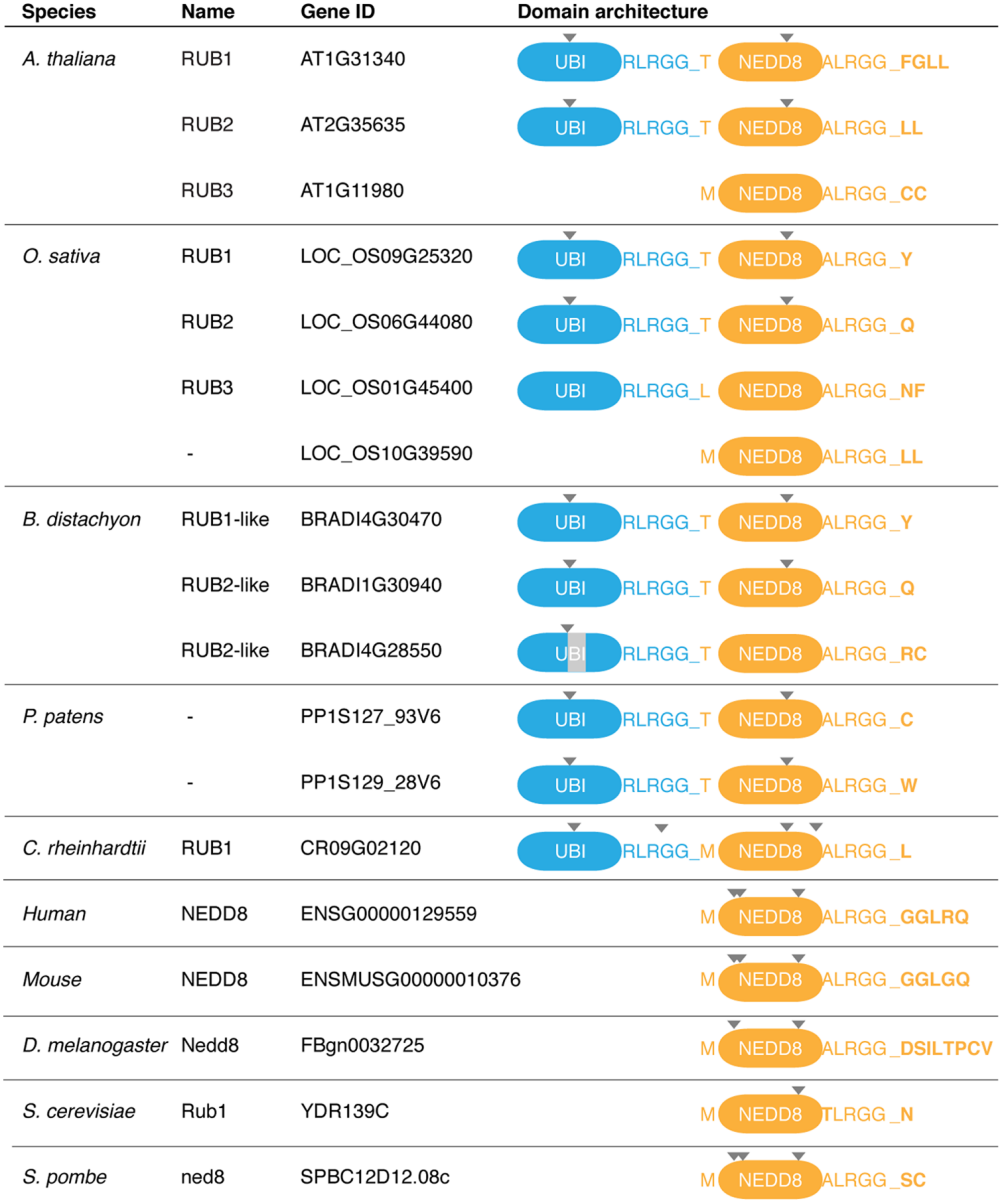
**Protein domain organization of NEDD8 proteins from several representative species.** Gene identification numbers (Gene IDs) are as listed in www.ensemblgenomes.org. Specifically indicated are the last five amino acids of the respective proteins before the proteolytic cleave sites, the first amino acid of NEDD8 and the proteins’ C-terminal amino acids. Proteolytic processing occurs after the C-terminal RGG residues and is indicated by an underscore. Positions of introns in the respective genes are indicated by a triangle. The light gray area in BRADI4G28550 highlights an apparent 22 amino acid deletion in the ubiquitin part of the protein. *A. thaliana* (*Arabidopsis thaliana*), *O. sativa* (*Oryza sativa*, rice), *B. distachyon* (*Brachypodium distachyon*), *P. patens* (*Physcomitrella patens*, moss), *C. reinhardtii* (*Chlamydomonas reinhardtii*, algae), *D. melanogaster* (*Drosophila melanogaster*, fruit fly), *S. cerevisiae* (*Saccharomyces cerevisiae*, baker’s yeast), *S. pombe* (*Schizosaccharomyces pombe*, fission yeast).

NEDD8 orthologs can be identified in all eukaryotic species that have sequenced genomes. While NEDD8 is a single gene in humans, mouse, and fruit fly, several copies of *NEDD8* are encoded by the genomes of the plant species *Arabidopsis* (*Arabidopsis thaliana*; Rao-Naik et al., 1998), rice (*Oryza sativa*)*, Brachypodium *(*Brachypodium distachyon*), and the moss *Physcomitrella patens *(**Figure 1**). All NEDD8 proteins require proteolytic processing of their C-termini to generate mature NEDD8 with a C-terminal glycine required for NEDD8 conjugation (**Figure 1**). An additional unique feature of plant NEDD8 is the existence of ubiquitin-NEDD8 gene fusions. While gene fusions of ubiquitin to ubiquitin itself or other genes have been reported in other species, *NEDD8* is an unfused gene in animals and yeasts but not in plants. This ubiquitin-NEDD8 fusion structure is found in *Arabidopsis* RUB1 and RUB2 and seems to be conserved among plants, mosses and algae (**Figure 1**; Rao-Naik et al., 1998; Vierstra and Callis, 1999; Shin et al., 2011). In RUB1 and RUB2, a single ubiquitin is fused head-to-tail to the N-terminus of NEDD8 and both ubiquitin-NEDD8 fusions then require post-translational processing to release monomeric ubiquitin and NEDD8 (**Figure 1**). Furthermore, plant genomes contain an unfused monomeric form of NEDD8, *RUB3* in *Arabidopsis*, that can additionally be distinguished from the other *RUB* genes because it lacks an intron that is present at a conserved position in other *RUBs*, e.g., in *Arabidopsis* *RUB1* and *RUB2* (**Figure 1**). The absence of an intron suggests that this less complex *RUB3* may be more ancient than the intron-containing *RUB1* or *RUB2* or that *RUB3* originated from an mRNA intermediate and a retrotransposition event (Huang et al., 2012).

Similarly to the high sequence conservation observed between human and *Arabidopsis* ubiquitin (96% amino acid sequence identity), also NEDD8 proteins are highly conserved between species (83% identity between human and *Arabidopsis*). This high level conservation is suggestive for an important function of NEDD8 conjugation (neddylation) in eukaryotic cells and a highly conserved neddylation and deneddylation machinery. Indeed, loss-of-*NEDD8* function causes lethality at an early developmental stage in most model organisms and also in plants, with the notable exception of *Saccharomyces cerevisiae* (Lammer et al., 1998; Liakopoulos et al., 1999; Jones and Candido, 2000; Osaka et al., 2000; Tateishi et al., 2001; Ou et al., 2002; Dharmasiri et al., 2003; Maytal-Kivity et al., 2003; Bostick et al., 2004). In *Arabidopsis*, not the single but the combined knockout of the genes *RUB1* and *RUB2* leads to a developmental arrest at the embryonic two-cell stage (Bostick et al., 2004). Thus, *NEDD8* genes and neddylation are essential for growth and development in plants. Plants with reduced *NEDD8* gene expression are dwarfed, partially insensitive to root growth inhibitory concentrations of the plant hormone auxin and also partially defective in auxin-induced lateral root formation (Bostick et al., 2004). As will be outlined below, auxin insensitivity phenotypes are reliable and at the same time the most obvious readouts of neddylation pathway mutants.

## NEDD8 PROCESSING

**FIGURE 2:**
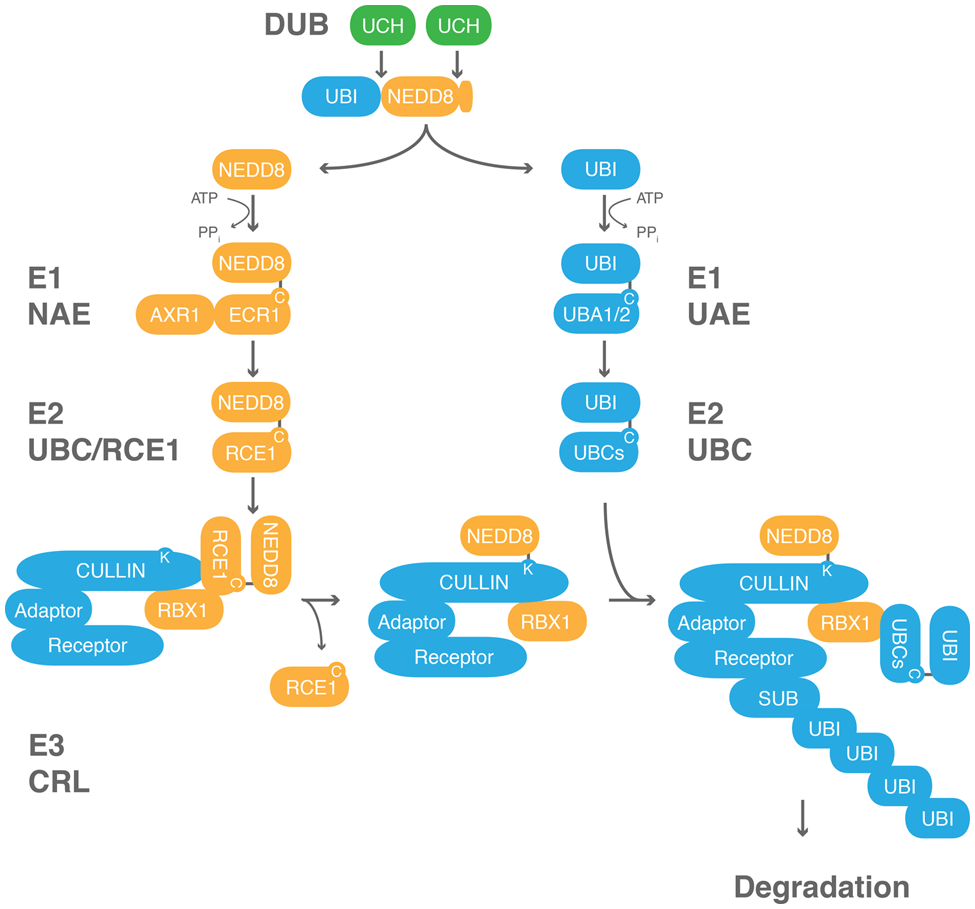
**Neddylation and ubiquitin modification are biochemically related processes.** Ubiquitin c-terminal hydrolases (UCHs) belong to the family of DUBs that process ubiquitin (UBI)-NEDD8 fusion proteins. UBI and NEDD8 are activated by their conjugation to E1 ubiquitin/NAEs. UBI or NEDD8 from the E1 are then passed via a transthiolation reaction to a protein of the family of E2 ubiquitin-conjugating enzymes, RUB1 CONJUGATING ENZYME1 (RCE1) in *Arabidopsis*. The ubiquitin-charged E2 can then form a complex with an E3 ubiquitin ligase, and ultimately, ubiquitin and NEDD8 are transferred to a lysine residue on the cullin of the E3 and the substrate, respectively. UBI but not NEDD8 can form chains. RBX1 and other proteins not shown here are the proposed NEDD8 ligases. Cullin-RING ligases (CRLs) are the superfamily of E3 ubiquitin ligases that are regulated by neddylation. CRLs are generally composed of a cullin subunit, RBX1, as well as a substrate (SUB) recognition module composed of an adaptor and a substrate receptor protein. C, cysteine; K, lysine.

NEDD8 is conjugated to the protein substrates via an isopeptide bond between its C-terminal glycine and a lysine of the target protein (**Figure 2**). NEDD8, like ubiquitin and most UBLs, is expressed as an inactive precursor with a short C-terminal extension that consists of one or several amino acids that need to be cleaved off to allow for NEDD8 conjugation (**Figures 1** and **2**; Jentsch and Pyrowolakis, 2000). It has been proposed that the C-terminal extension of ubiquitin, NEDD8, and other UBLs serves to prevent unprocessed proteins to enter into the conjugation pathway but there is, in fact, no experimental evidence supporting this hypothesis (Callis et al., 1995; Rao-Naik et al., 1998). The plant ubiquitin-NEDD8 fusion proteins additionally require removal of the N-terminal ubiquitin by proteolytic cleavage.

**FIGURE 3:**
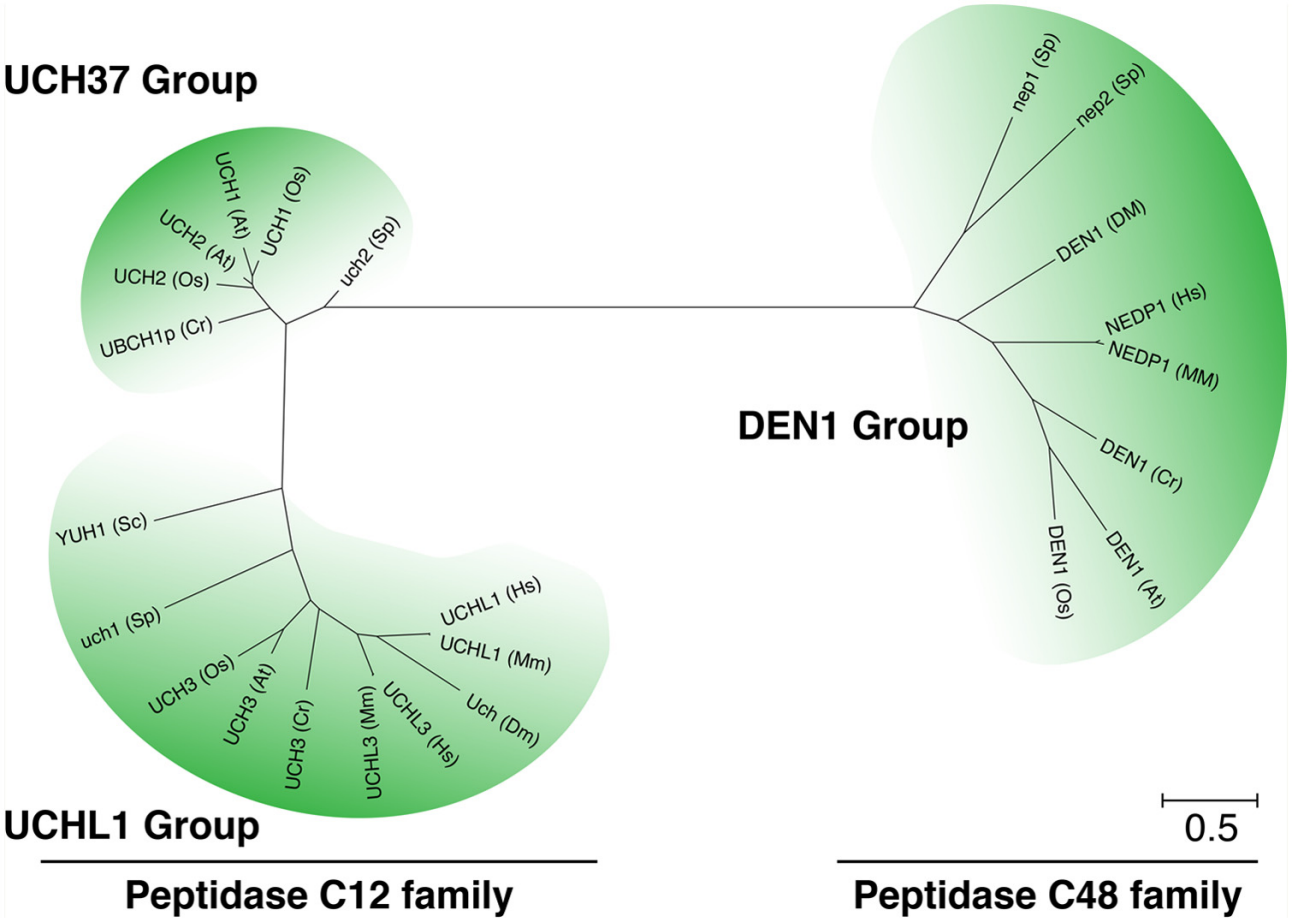
**NEDD8 processing is mediated by at least three different classes of peptidases.** Unrooted phylogenetic tree of representative members of the UCH and DEN1/NEDP1/SENP8 protein families from *Arabidopsis thaliana* (At), rice (*Oryza sativa*, Os), algae (*Chlamydomonas reinhardtii*, Cr), baker’s yeast (*Saccharomyces cerevisiae*, Sc), fission yeast (*Schizosaccharomyces pombe*, Sp), fruit fly (*Drosophila melanogaster*, Dm), mouse (*Mus musculus*, Mm), and human (*Homo sapiens*, Hs). Molecular phylogenetic analysis was performed based on the Maximum Likelihood method and the JTT matrix-based model (Jones et al., 1992) using CLUSTALW algorithm at the EMBL-EBI website (http://www.ebi.ac.uk/Tools/msa/clustalw2/). The tree with the highest log likelihood (-5916.8263) is shown. Initial tree(s) for the heuristic search were obtained automatically by applying the Maximum Parsimony method. The tree is drawn to scale, with branch lengths measured in the number of substitutions per site. The analysis involved 24 protein sequences. All positions containing gaps and missing data were eliminated. There were a total of 145 positions in the final dataset. Evolutionary analyses were conducted in MEGA5 (Tamura et al., 2011).

NEDD8 processing is carried out by ubiquitin C-terminal hydrolases (UCH) from the family of deubiquitinating enzymes (DUBs). In *S. cerevisiae* and humans, NEDD8 precursor C-terminal processing is facilitated by a dual specificity UCH of the C12 family peptidases, Yuh1 (yeast) or UCHL3 (human, mouse), which also processes the C-terminal extensions of ubiquitin (**Figure 3**; Wada et al., 1998; Johnston et al., 1999; Linghu et al., 2002; Hemelaar et al., 2004; Frickel et al., 2007; Yu et al., 2007). To date, the only isopeptidase known to function exclusively in NEDD8 processing and deconjugation is the C48 family peptidase DEN1/NEDP1/SENP8 from *Drosophila, *and human (**Figure 3**; Gan-Erdene et al., 2003; Mendoza et al., 2003; Wu et al., 2003; Shen et al., 2005; Chan et al., 2008; Shin et al., 2011). However, mouse knockouts of *UCHL3* or *Drosophila* and *Aspergillus* knockouts of *DEN1* are viable although NEDD8 and neddylation are essential in the respective organisms (Kurihara et al., 2000; Chan et al., 2008; Christmann et al., 2013). These findings suggest that mutants of these processing enzymes cannot be fully impaired in NEDD8 processing.

In non-plant species where NEDD8 is expressed as an unfused gene, examining NEDD8 precursor processing *in vivo* is not trivial because NEDD8 has only a short C-terminal extension. Therefore, conjugation of processed NEDD8 to cullins is used as an indirect indication for proper processing. Since cullin neddylation was not impaired in any of the *UCH* gene mutants examined to date, it can be inferred that also NEDD8 processing and conjugation are at least partially functional in these mutants (Kurihara et al., 2000; Chan et al., 2008; Christmann et al., 2013). In the fission yeast *Schizosaccharomyces pombe*, even a *Δnep1 Δnep2 Δuch1 Δuch2* quadruple mutant lacking the two C48 peptidases orthologous to DEN1 (*NEP1* and *NEP2*) as well as the two C12 peptidases orthologous to yeast Yuh1 (*UCH1* and *UCH2*) shows efficient cullin neddylation (O’Donoghue et al., 2013). Only the additional knockout of the cullin deneddylating enzyme COP9 SIGNALOSOME SUBUNIT5 (CSN5) hinted to a reduced efficiency of NEDD8 precursor processing in this complex mutant because it revealed the absence of cullin1 hyperneddylation that can be observed in the *Δcsn5* deletion strain (O’Donoghue et al., 2013). Taken together, these various findings suggest that there is a functionally redundant family of NEDD8 processing enzymes and that there may be other as yet unknown peptidases that also participate in NEDD8 processing. Furthermore, it is possible that there is no strict specificity among the ubiquitin and NEDD8 processing enzymes *in vivo*.

### NEDD8 PROCESSING IN PLANTS

NEDD8 processing hydrolases from plants remain to be identified. In *Arabidopsis*, several peptidases can be classified as C12 and C48 family peptidases based on their homology to UCHs from other species (**Figure 3**). To date, only the C12 peptidases UCH1 and UCH2 have been analyzed at the biological level (Yang et al., 2007). Based on their homology to C12 peptidases, these UCHs would be predicted to have a role in ubiquitin processing. However, although both UCH proteins showed the predicted ubiquitin processing activity* in vitro*, neither the *uch1 uch2* double mutant nor *UCH1* overexpressing lines had apparent changes in the pattern of ubiquitin conjugate formation or in the abundance of free monomeric ubiquitin. At the phenotypic level, mutants or overexpression lines display impaired shoot and flower development and changes in the rate of leaf formation. Altering the abundance of the two *UCH* genes also affects auxin and cytokinin responsiveness. These phenotypes may be explained by defects in selective rather than general protein degradation and this hypothesis is supported by the observation that the degradation of the auxin-labile AUX/IAA AUXIN RESISTANT3 (AXR3) but not that of the light-labile phytochrome A or ELONGATED HYPOCOTYL5 (HY5) proteins appeared to be affected in the *uch* mutants. In summary, these findings suggest that UCH1 and UCH2 may not only act at the level of ubiquitin processing but may also act by selectively regulating the proteasomal degradation of proteins by antagonizing substrate ubiquitylation. Whether UCH1 and UCH2 have a role in NEDD8 processing remains to be examined but the comparatively weak morphological phenotype as well as the absence of an apparent cullin neddylation phenotype already suggests that the two UCH proteins may not have a major function in this process.

## NEDDYLATION

NEDD8 is conjugated to target proteins in a manner that is highly similar to ubiquitin conjugation (**Figure 2**). NEDD8 is activated by an E1 NEDD8 activating enzyme (NAE) and then passed on to an E2 NEDD8 conjugating enzyme of the ubiquitin-conjugating (UBC) enzyme family from where the protein is ultimately transferred to its substrate protein. The best-studied NEDD8 conjugates are the cullin subunits of cullin-RING-type E3 ubiquitin ligases (CRLs; Hua and Vierstra, 2011). CRLs are a family of evolutionarily conserved E3 ligases that are composed of a core complex, comprised of a cullin subunit and the RING BOX PROTEIN1 (RBX1), as well as a ubiquitylation substrate recognition module. In plants, three different types of CRL complexes can be distinguished based on the identity of the cullin subunits CULLIN1, CULLIN3, or CULLIN4 and the identity of their respective substrate recognition module (Lammer et al., 1998; Ruegger et al., 1998; Dieterle et al., 2005; Figueroa et al., 2005; Gingerich et al., 2005; Bernhardt et al., 2006; Chen et al., 2006). The CRL subunit RBX1 is common to all CRLs and serves to promote NEDD8 conjugation.

Structural analyses of cullin neddylation revealed that NEDD8 conjugation causes a conformational change in subdomains of the cullin and RBX1 subunits (Duda et al., 2008; Boh et al., 2011). Neddylation also eliminates the binding of the exchange factor CULLIN-ASSOCIATED-NEDD8-DISSOCIATED1 (CAND1) and locks the CRL in an active state. Thus, neddylation controls CRL activity by promoting conformational changes that favor substrate ubiquitylation. CRL neddylation can then also lead to the recruitment of additional regulatory factors (den Besten et al., 2012).

### THE NEDDYLATION PATHWAY AND AUXIN INSENSITIVITY

As will be discussed in more detail below, loss of cullin neddylation or cullin deneddylation affect CRL function by promoting or, respectively, preventing interactions with the substrate receptor exchange factor CAND1. In the plant and neddylation biology context, the *Arabidopsis* CULLIN1-containing E3 ligase SCF^TIR1^ with the substrate recognition module composed of the F-box protein (FBP) TRANSPORT INHIBITOR RESISTANT1 (TIR1) and its adaptor subunit *ARABIDOPSIS* SKP1 (ASK) is highly relevant (Ruegger et al., 1998; del Pozo and Estelle, 1999). TIR1, functioning at the same time also as an auxin receptor, binds AUX/IAA transcriptional repressors in an auxin-dependent manner and targets AUX/IAAs for ubiquitylation and degradation by the 26S proteasome (Gray et al., 2001; Tan et al., 2007). AUX/IAAs and auxin-induced AUX/IAA degradation regulate a number of important developmental and morphological processes throughout plant development: *bodenlos* (*bdl*) mutants expressing a stabilized (non-degradable) variant of the AUX/IAA protein bodenlos/iaa14 (bdl/iaa14) are deficient in embryonic root differentiation and are consequently rootless (Hamann et al., 1999; Weijers et al., 2005). *axr3* mutants express the stabilized axr3/iaa17 protein and this mutation allows for root elongation in the presence of root growth-inhibiting auxin concentrations (Gray et al., 2001). The link between auxin insensitivity (auxin resistance), AUX/IAA degradation and CULLIN1 could be established because the CULLIN1 alleles *axr6-1* and *axr6-2* were identified based on their auxin insensitivity (Hobbie et al., 2000; Shen et al., 2002; Hellmann et al., 2003; Quint et al., 2005; Esteve-Bruna et al., 2013). While homozygous *axr6-1* and *axr6-2* loss-of-function mutants arrest development during embryogenesis, the heterozygous**mutants display the auxin-insensitive root growth elongation phenotype. Furthermore, double mutants of *axr6-1* or *axr6-2* with other mutants of the auxin and neddylation pathway are defective in root differentiation and thereby mimic the characteristic phenotype of the *bdl* mutant (Hobbie et al., 2000; Hellmann et al., 2003; Quint et al., 2005; Esteve-Bruna et al., 2013).

### NEDD8 ACTIVATION

Both, defects in root differentiation as well as auxin-insensitive root elongation have been used extensively as phenotypes for the identification and characterization of *Arabidopsis* neddylation mutants. *auxin resistant1* (*axr1*) is a mutant of the NAE enzyme AXR1 and was identified due to its defects in auxin response that could later be explained by impairment in the degradation of the AUX/IAA protein AXR3 (Lincoln et al., 1990; Leyser et al., 1993; del Pozo et al., 1998). In *Arabidopsis*, *axr1* mutants display an auxin-insensitive root growth phenotype but to fully impair NEDD8 conjugation the function of the AXR1-paralog *AXR1-LIKE* (*AXL*) also needs to be deleted (Dharmasiri et al., 2007). *axr1 axl1* double mutants have a more severe phenotype than *axr1* mutants in that they are defective in embryonic root differentiation and mimic the *bdl* mutant phenotype (Dharmasiri et al., 2007). *Arabidopsis* AXR1 and AXL proteins appear to be equivalent at the biochemical level but interestingly have a differential ability to complement the *axr1* mutant phenotype when expressed from the *AXR1* promoter (Dharmasiri et al., 2007; Hotton et al., 2011). While NEDD8 activation is carried out by a single protein in animals and yeasts, NAE is a heterodimer in plants of AXR1/AXL and E1 C-TERMINAL RELATED1 (ECR1) corresponding to the protein’s N- and C-termini, respectively (**Figure 2**; del Pozo et al., 1998; Hotton et al., 2011). An *ecr1-1* mutant was identified in a screen for mutants with differential auxin sensitivity and *axr1/axl* mutants as well as *ecr1* mutants are defective in cullin neddylation (Woodward et al., 2007).

### NEDD8 CONJUGATION

RUB1 CONJUGATING ENZYME1 (RCE1) was identified in *Arabidopsis* based on its homology to human UBC12 (del Pozo and Estelle, 1999). An *rce1-1* insertion mutant with significantly reduced *RCE1* expression levels was subsequently isolated and found to be strongly impaired in cullin neddylation (Dharmasiri et al., 2003). *rce1-1* mutants display auxin insensitive root growth phenotypes and fail to differentiate a primary root when combined with *axr1*. Two additional *rce1* alleles were recently found in a suppressor screen of the auxin overproducing *sur2* mutant (Pacurar et al., 2012). Interestingly, both of these *rce1* alleles would be expected to interfere significantly with the biochemical activity of RCE1 since they carry a nonsense and splice site mutation in exon 4, respectively. Analyzing the extent to which RCE1 function is affected in these alleles is certainly interesting because the unexpectedly weak phenotype of these supposedly strong alleles could be considered indicative for the existence of functionally redundant NEDD8 conjugating enzymes.

### NEDD8 LIGATION

The CRL core subunit RING BOX1 (RBX1) is one candidate for an E3 NEDD8 ligase (Morimoto et al., 2003). RBX1 is encoded by two genes in *Arabidopsis* and its function as CRL subunit and as NEDD8 ligase was addressed in mutants, antisense and overexpression lines (Gray et al., 2002; Lechner et al., 2002; Schwechheimer et al., 2002). RBX1 interacts with RCE1 and while cullin neddylation is decreased in the absence of *RBX1 *it is increased when *RBX1* is overexpressed (Gray et al., 2002).

The protein DEFECTIVE IN CULLIN NEDDYLATION1 (DCN1) has also been described as an E3 NEDD8 ligase (Kurz et al., 2005; Kurz et al., 2008; Meyer-Schaller et al., 2009). Based on yeast studies, it has recently been proposed that DCN1 increases the substrate specificity of RBX1 by directing the RBX1-bound NEDD8-E2 toward the cullin (Scott et al., 2010). Additionally, it was shown that the interaction between DCN1 and UBC12 is regulated by the N-terminal acetylation of the UBC12 E2 enzyme (Scott et al., 2011). The analysis of DCN1-LIKE proteins, of which there are five in humans, has also revealed that at least one member of the protein family, DCNL3, is bound to the plasma membrane (Meyer-Schaller et al., 2009). Studies of a mammalian CULLIN2-containing CRL further revealed that DCN1-LIKE1 can engage in interactions between the cullin and the respective substrate receptor subunit, and more importantly, that this interaction is strengthened when the substrate receptor is loaded with cargo (Heir et al., 2013). At least in this case, DCN1-LIKE1 may function as a sensor for degradation substrate availability and consequently promote neddylation. Thus, DCN1 proteins may contribute to the regulation of E3 ligase activity by targeting E3 ligases to or by activating them in specific subcellular locations.

As yet, DCN1 or DCN1-LIKE proteins have not been analyzed in plants but AT3G12760 is a candidate for a direct DCN1 ortholog from *Arabidopsis*. A second DCN1-LIKE protein, less closely related to DCN1 than AT3G12760, was identified as *anti-auxin resistant3* (*AAR3*) in a screen for mutants that showed resistance to the anti-auxin *p*-chlorophenoxyisobutylic acid. The same screen also identified mutant alleles of *TIR1* and *CULLIN1* and, based on the shared phenotype of these mutants, AAR3/DNC1-LIKE would qualify as a candidate regulator of NEDD8 ligation and SCF^TIR1^ function (Biswas et al., 2007). Unfortunately, the biochemical function of AAR3/DNC1-LIKE in the context of cullin neddylation has not been examined as yet. There is also one further *Arabidopsis* gene that may encode for an additional DCN1-LIKE protein. Thus, the biochemical and biological functions of *DCN1-LIKE* genes from *Arabidopsis* remain to be investigated.

In yeast, also TFB3, a RING domain subunit of the general transcription factor TFIIH was found to promote neddylation in addition to RBX1 and DCN1 (Rabut et al., 2011). The identification of TFB3 was based on initial observations that CULLIN4 neddylation in yeast was independent from RBX1 and DCN1. The analysis of RING domain protein mutants from yeast then led to the subsequent discovery of Tfb3 as a RING domain protein responsible for the neddylation of CULLIN3 and CULLIN4. A clear homolog of TFB3 is not easily discernable in the plant genomes but the identification of TFB3 from yeast *per se* indicates that it cannot be ruled out that besides RBX1 and DCN1 also other NEDD8 ligases exist in plants.

### MLN4924 – A NEDDYLATION INHIBITOR

The importance of the NEDD8-modification pathway in the control of plant development has recently been elucidated in a study with the neddylation inhibitor MLN4924 (Hakenjos et al., 2011). MLN4924 was initially described as an inhibitor of the human NAE E1 enzyme but was subsequently found to also inhibit the NAE E1 subunit ECR1 from predictably all plant species (Soucy et al., 2009; Brownell et al., 2010; Hakenjos et al., 2011). MLN4924 inhibits neddylation in plants and the impairment of CRL function results in the degradation of a number of CRL substrates such as the AUX/IAAs of the auxin pathway, DELLA proteins of the gibberellin pathway and the cell cycle regulator KRP1 (Hakenjos et al., 2011). While the severe phenotypes of strong NEDD8 pathway mutants in *Arabidopsis* and the absence of neddylation mutants in other plant species has as yet hampered studying the role of neddylation in all stages of plant development or in non-*Arabidopsis* species, the availability of MLN4924 now overcomes this limitation (Hakenjos et al., 2011).

### NEDDYLATION MUTANTS ARE IMPAIRED IN MANY DIFFERENT CRL FUNCTIONS

As outlined above, AUX/IAA degradation is partially or fully impaired in all mutants of the NEDD8 conjugation pathway and auxin responses are partially or fully blocked in these mutant backgrounds. However, the phenotype of the NEDD8 conjugation mutants is much more complex and not only the consequence of defects in the auxin response pathway. In this regard, it is important to realize that plants predictably have many hundreds of CRLs and that all these CRLs should be impaired in neddylation mutants (Xu et al., 2009). Among these CRLs, SCF^TIR1^ and closely related complexes implicated in auxin responses have a very prominent role because defects in the auxin-regulatory CRLs lead to morphological defects that can easily be examined (Dharmasiri et al., 2005a,b). However, while malfunction of SCF^TIR1^ and closely related complexes is the most visible phenotype of neddylation mutants, all CRL functions should be affected in *axr1* mutants in a manner similar to the defects observed in the auxin pathway. This fact is sometimes overlooked and particularly *axr1* mutants that were amongst the first auxin response mutants to be identified (Lincoln et al., 1990; Leyser et al., 1993) are often being used as auxin pathway-specific mutants. The knowledge about the existence of many other CRL-dependent pathways, also CRL pathways that affect plant growth and morphology clearly argue against using *axr1* mutants or other neddylation mutants as auxin pathway-specific mutations for morphological analyses or genetic interaction studies (Dill et al., 2004; Shen et al., 2007; Stirnberg et al., 2007; Nelson et al., 2011; Waters and Smith, 2013).

## CSN PROMOTES CULLIN DENEDDYLATION

**FIGURE 4:**
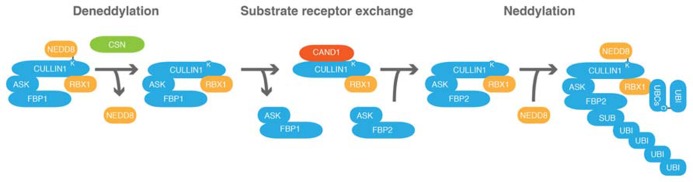
**CAND1 regulates the cells CRL repertoire by promoting the exchange of substrate receptor subunits.** Schematic representation of the exchange of a hypothetical F-BOX PROTEIN1 (FBP1) of the CRL SCF^FBP1^ against FBP2 following CULLIN1 deneddylation. *ARABIDOPSIS* SKP1 (ASK) proteins are adaptor subunits that link FBPs with CULLIN1. CULLIN1, ASK, RBX1 and FBP form an SCF-type CRL. COP9 SIGNALOSOME (CSN) promotes CULLIN deneddylation. CULLIN-ASSOCIATED-NEDD8-DISSOCIATED1 (CAND1) is an exchange factor that can only associate with deneddylated cullins and promotes substrate receptor exchange. C, cysteine; K, lysine.

NEDD8 can be deconjugated from CRLs through the activity of the COP9 signalosome (CSN; **Figure 4**). CSN is evolutionarily conserved and in most species including plant and mammalian species composed of eight subunits (Chamovitz et al., 1996; Seeger et al., 1998; Wei and Deng, 1998). CSN was originally identified in plants based on mutants that display a constitutively photomorphogenic (cop) phenotype and named following the identification of the causative mutation in the *cop9* mutant (Wei and Deng, 1992; Wei et al., 1994). Similarly to light-grown seedlings, *cop* mutants have a short hypocotyl, open cotyledons, and express light-regulated genes when grown in the dark.

### CSN REPRESSES PHOTOMORPHOGENESIS IN *ARABIDOPSIS*

In *Arabidopsis*, loss-of-function of the eight CSN subunits results in most cases in the destabilization of the entire CSN complex and in a phenotypically indistinguishable *cop* phenotype, marking constitutive photomorphogenesis as a hallmark phenotype for loss of CSN function (Serino et al., 1999, 2003; Dohmann et al., 2005; Gusmaroli et al., 2007). The *cop* phenotype of *csn* mutants can be explained by their inability to degrade photomorphogenesis regulatory transcription factors such as HY5 in dark-grown seedlings through the activity of the E3 ligase COP1 (Osterlund et al., 2000; Chen et al., 2006; Lau and Deng, 2012). COP1 function is impaired in *csn* mutants leading to a stabilization of the COP1 targets also in the dark. In the wildtype, photomorphogenic development during germination is seemingly controlled by the light-controlled nucleocytoplasmic shuttling of COP1 (von Arnim and Deng, 1994; Osterlund et al., 2000; Pacin et al., 2013). In contrast to the strong photomorphogenesis phenotype of *csn* loss-of-function mutants, mutants with partially impaired CSN function display a number of phenotypes, also including auxin insensitive root elongation (Schwechheimer et al., 2001, 2002; Dohmann et al., 2005, 2008b; Stuttmann et al., 2009; Huang et al., 2013b). This phenotypic similarity was indicative for a connection between CSN, the neddylation pathway, and SCF^TIR1^-dependent plant growth regulation when knowledge about the biochemical interplay of these components was still unclear (Schwechheimer et al., 2001).

### CULLIN DENEDDYLATION IS A FUNCTION OF THE MPN+ DOMAIN SUBUNIT CSN5

COP9 signalosome is closely related to the “lid” of the 26S proteasome. In plants and animals, both protein complexes are composed of six so-called PCI domain subunits and two MPN-domain subunits and they share a set of subunit–subunit interactions within the respective complexes (Glickman et al., 1998; Wei et al., 1998; Fu et al., 2001; Enchev et al., 2010; Kotiguda et al., 2012). The relatedness of the two complexes and their in part shared biochemical function are nicely reflected by the fact that a proteasomal “lid” subunit functionally replaces a “missing” CSN subunit in *Saccharomyces cerevisiae *(Yu et al., 2011). CSN as well as the “lid” have two MPN-domain proteins, which can be further subdivided into an MPN+ domain protein with a catalytically active metalloprotease site, and a catalytically inactive MPN-domain protein that must be derived from the MPN+-domain counterpart (Maytal-Kivity et al., 2002). The MPN+ domain subunits CSN5 and RPN11 confer deneddylation and deubiquitylation activity to the CSN and proteasome “lid” complexes, respectively (Cope et al., 2002; Ambroggio et al., 2004). *csn* mutants from *Arabidopsis* are fully impaired in cullin deneddylation and only traces of, presumably *de novo* synthesized, unneddylated cullin can be detected in *csn* mutants. Interestingly, CSN5 is only functional as a cullin deneddylase when associated with CSN. CSN physically interacts with the cullin and RBX1 subunits of CRLs through its subunits CSN2 and CSN6 and it is thought that these interactions provide CSN with the affinity for its CRL targets (Schwechheimer et al., 2001). Interesting is also the recently identified *csn3-3* allele, which carries a missense mutation in the *CSN3* gene. This mutation strongly impairs auxin responses in the mutant but does neither obviously affect cullin deneddylation nor CSN protein complex integrity (Huang et al., 2013a). Thus, the affected domain of CSN3 may be required for an as yet unknown essential CSN function such as CRL subunit interactions or for the ability of the protein or protein complex to engage in other interactions required for normal auxin responses. This is supported by biochemical analyses combined with structural electron microscopy that suggest that CSN2 and CSN5 interact with the cullin E3 ligase subunit whereas F-box substrate receptors interact with CSN1 and CSN3 (Enchev et al., 2012).

Since *csn* mutants are impaired in the function of presumably hundreds of E3 ligases, it is not surprising that additional physiological defects have been identified in these mutants that can be explained by defects in other CRLs and include defects in SCF^COI1^-mediated jasmonate signaling (Schwechheimer et al., 2002; Hind et al., 2011), SCF^SLY1^-mediated gibberellin signaling (Dohmann et al., 2010) as well as defects in cold response (Schwechheimer et al., 2002), cell cycle progression (Dohmann et al., 2008a), and the control of ascorbic acid synthesis (Wang et al., 2013).

### CSN REGULATION AND CSN REGULATORS

In view of the large number of CRLs that exist in eukaryotic cells and the importance of CSN-dependent cullin deneddylation for CRL function, it has to be asked how CRL neddylation and CSN-dependent deneddylation are regulated. In this context, it was shown for different CRLs that the availability of ubiquitylation substrate receptor and ubiquitylation substrate promotes CRL formation and cullin neddylation (Bornstein et al., 2006; Emberley et al., 2012). The association of FBP-SKP1 dimers can inhibit CSN function on selected SCF-complexes several fold (Emberley et al., 2012). Thus, in the case of SCF-complexes cullin neddylation and deneddylation are regulated by the presence of FBP-SKP1 dimers and particularly by the presence of a given FBP, assuming that there is no regulation on the level of the SKP1 adaptor subunit, which does not confer substrate specificity to the SCF complexes. CSN is even more strongly inhibited in the presence of degradation substrate and thus degradation substrate and degradation substrate receptor availability negatively regulate CSN activity (Emberley et al., 2012). Furthermore, CSN associates tightly with deneddylated SCF and CSN thereby keeps the CRL complex in a state of low activity after substrate degradation (Emberley et al., 2012). Through this interaction, CSN also prevents cullin neddylation, unless binding of a ubiquitylation substrate triggers its dissociation and allows for cullin neddylation (Enchev et al., 2012).

In addition to the regulation of CSN by CRLs, their subunits and their substrates, there are also other candidate regulators whose function remains to be determined. The 7 kDa protein SMALL ACIDIC PROTEIN1 (SMAP1) is an interesting CSN-interaction partner (Nakasone et al., 2012). *smap1* mutants were identified as *anti-auxin resistant1* (*aar1*) in a mutant screen that also identified mutants of *TIR1*, *CULLIN1*, and *DCN1-LIKE*. Thus the *aar1*/*smap1* phenotype may well be explained by a defect in the E3 ligase SCF^TIR1^ or its neddylation or deneddylation. Importantly, immunoprecipitates of SMAP1 are very strongly enriched in at least six CSN subunits, indicating that SMAP1 is a CSN interactor and may regulate CSN function. As yet, the analysis of CULLIN neddylation patterns did not reveal any apparent defects in its neddylation or deneddylation but the *aar3*/*smap1* mutant phenotype together with the SMAP1-CSN interaction strongly suggests that SMAP1 is linked to CSN function. Since there is not apparent homolog of SMAP1 outside of the plant kingdom, this function should be plant specific.

The analysis of the CSN-interacting Rig-G protein, a protein related to the *Arabidopsis* protein SPINDLY, provided some insights into how interaction partners could interfere with CSN activity. In the case of Rig-G it is proposed that the protein recruits CSN subunits to the cytoplasm and thereby interferes with CSN assembly in the nucleus (Xu et al., 2013).

## CAND1 – A SUBSTRATE RECEPTOR EXCHANGE FACTOR FOR CRLs

Important progress has been achieved in the understanding of the role of neddylation and deneddylation of cullins in the context of CRL assembly and function through analysis of the protein Cullin-associated-Nedd8-dissociated-1 (CAND1). As its name already reveals, CAND1 was identified as an interactor of non-neddylated cullins (Liu et al., 2002; Zheng et al., 2002; Oshikawa et al., 2003). Through a series of elegant experiments from at least three independent laboratories it was recently shown that CAND1 functions as a novel type of exchange factor for CRLs (Pierce et al., 2013; Wu et al., 2013; Zemla et al., 2013). In a highly quantitative and not only therefore remarkable analysis of the diverse protein–protein interactions that can take place between the subunits of SCF-type CRL complexes and CAND1, it could be shown that CAND1 can promote the disassembly of SCF complexes and that FBPs can remove CAND1 from CULLIN1 (Pierce et al., 2013; **Figure 4**). When testing 21 different FBPs it was found that 20 of these could be exchanged using CAND1 as an exchange factor. Thus, in the case of SCF-type CRLs and most likely also in the case of CRLs that are formed with the other cullins, CAND1 can modulate the CRL-complex repertoire of the cell.

CAND1 is unable to interact with neddylated cullins and cullin neddylation stabilizes specific CRLs to prevent substrate receptor exchange (Emberley et al., 2012). Upon cullin deneddylation, CAND1 can become active and modulate the CRL repertoire to optimally match substrate receptor demand. Thus, there must be mechanisms to control CRL deneddylation. Indeed, cullin neddylation and deneddylation are controlled by the presence and absence of degradation substrates or their interactions with substrate receptors (Bornstein et al., 2006; Chew and Hagen, 2007; Emberley et al., 2012). Furthermore, CSN binds preferentially to neddylated CRLs, which may also recruit CSN-associated proteins important for CRL regulation (den Besten et al., 2012). CSN can bind deneddylated cullins but is dissociated in the presence of degradation substrate receptors and degradation substrates (Choo et al., 2011). Consequently, it can be inferred that *csn* as well as *cand1* mutants are deficient in releasing specific substrate receptors. This hypothesis could be experimentally confirmed and it could be shown that substrate receptor activation and substrate degradation are delayed in such mutant backgrounds (Zemla et al., 2013).

### CAND1 IN PLANTS

In plants, *cand1* mutants were identified and analyzed in reverse and forward genetic screens. Mutants deficient in CAND1 were isolated as auxin-resistant mutants and the mutant spectrum of *cand1* mutants was recognized as being highly similar to, but also to exceed that of *axr1* mutants (Cheng et al., 2004; Chuang et al., 2004; Feng et al., 2004). Three further *cand1* alleles were identified based on mutants with severe defects in leaf vein patterning (Alonso-Peral et al., 2006). In rice, CAND1 is required for the formation of crown roots and defects in crown root formation are associated with a cessation in the G2/M phase progression in these mutants (Wang et al., 2011). In this context it is interesting to note that also *Arabidopsis* *csn* mutants are defective in G2/M phase progression (Dohmann et al., 2008a).

*Arabidopsis* CAND1 also preferentially binds to non-neddylated cullins (Feng et al., 2004). Importantly, two sets of weak *Arabidopsis* mutants exist, the semi-dominant *axr6-1* and *axr6-2* on the one side and the recessive *cul1-6* on the other, carrying missense mutations in almost adjacent positions of CULLIN1. Interestingly, the respective mutant proteins interact differentially with CAND1. While the cul1-6 protein is deficient in CAND1 interaction, axr6-1 and axr6-2 bind more strongly to CAND1 (Feng et al., 2004; Moon et al., 2007). The availability of mutants with weak and strong defects in cullin function, cullin deneddylation, and CAND1 interaction has already permitted to assay the biochemical interactions of the various components at the genetic level (Zhang et al., 2008).

## NEDDYLATION SUBSTRATES

### EVIDENCE FOR NON-CULLIN NEDDYLATION SUBSTRATES

Despite extensive research, the role and importance of neddylation in cellular processes besides the regulation of CRL activity remains poorly understood. Contrary to the expanding knowledge about ubiquitylated proteins in eukaryotes including plants (Kim et al., 2013) similar studies for NEDD8 have so far not succeeded in consistently identifying non-cullin neddylated proteins (Li et al., 2006; Norman and Shiekhattar, 2006; Jones et al., 2008; Xirodimas, 2008; Bennett et al., 2010; Hakenjos et al., 2011; Hotton et al., 2012). However, there is evidence for the existence of a broad range of neddylated proteins and several non-cullin neddylated proteins have already been identified as summarized in **Table 1**. Loss of function mutants of DEN1/NEDP1/SENP8 from three different species, namely fruit fly, *Schizosaccharomyces pombe* and *Aspergillus nidulans* accumulate neddylated proteins over a broad range of molecular weights. At the same time, these mutants do generally not accumulate neddylated cullins suggesting that DEN1/NEDP1/SENP8 is an important deneddylating enzyme of these non-cullin neddylation substrates (Zhou and Watts, 2005; Chan et al., 2008; Christmann et al., 2013). Also overexpression of NEDD8 leads to the apparent enrichment of many neddylated proteins and this neddylation can be blocked with the inhibitor MLN4924 (Hakenjos et al., 2011). Thus, there is evidence that NEDD8-modified proteins other than the cullins can exist but may be low in abundance or only transiently modified under normal conditions.

**Table 1:** Neddylation substrates.

Neddylated protein	Proposed function of neddylation	Species	Reference
**E3 ubiquitin ligases**
Cullins, Cul7, and PARC	Increases activity	Eukaryotes	Hori et al. (1999), Sarikas et al. (2011), Calabrese et al. (2011)
Mdm2	Decreases activity	Human	Xirodimas et al. (2004)
Parkin	increases activity	Human	Um et al. (2012), Choo et al. (2012)
BRAP2	–	Human	Takashima et al. (2013)
pVHL	Changes pVHL protein interaction	Human	Stickle et al. (2004), Russell and Ohh (2008)
DIAP1/XIAP	-	Fruit fly/human**	Broemer et al. (2010)
DDB1	-	*Arabidopsis*	Hotton et al. (2012)
**Transcription factors**
p53	Inhibits transcriptional activity	Human	Xirodimas et al. (2004), Abida et al. (2007)
p73	Inhibits transcriptional activity by sequestering Tap73 to the cytoplasm	Human	Watson et al. (2006)
AICD	Inhibits transcriptional activity	Human	Lee et al. (2008)
E2F1	Inhibits transcriptional activity by blocking protein interaction	Human	Aoki et al. (2012), Loftus et al. (2012)
HIF1α	Stabilizes protein	Human	Ryu et al. (2011)
**Transcriptional inhibitors**
BCA3	Activates by promoting protein interaction	Human	Gao et al. (2006)
RCAN1	Stabilizes by inhibiting proteasomal degradation	Human	Noh et al. (2012)
**Receptors**
EGFR	Promotes receptor ubiquitylation and ligand induced degradation	Mammals	(Oved et al., 2006)
TβRII	Stabilizes protein	Human	(Zuo et al., 2013)
**Kinases**
PINK1	Stabilizes the cytosolic protein form	Human	Choo et al. (2012)
CK1α	–	Human	Huart et al. (2012)
**Other**
L11, S14, and other ribosomal proteins	Stabilizes the protein	Human	Xirodimas et al. (2008), Zhang et al. (2012)
SHC	–	Human	Jin et al. (2013)
HUR	Stabilizes the protein	Human	Embade et al. (2012)
Histone H4	Induces complex formation and amplifies Ubi cascade	Human	Ma et al. (2013)
drICE/caspase 7	Reduces catalytic activity	Fruit fly/human	Broemer et al. (2010)
Lag2	–	Yeast	Siergiejuk et al. (2009)
ML3	–	*Arabidopsis*	Hakenjos et al. (2013)

*PARK, Parkin-like cytoplasmic protein; Mdm2, murine double minute 2; Parkin, Parkinson juvenile disease protein 2; BRAP2, BRCA1-associated protein; DIAP1, *Drosophila *inhibitor of apoptosis 1; XIAP, X-linked inhibitor of apoptosis protein; DDB1, damaged DNA binding protein1; VHL, Von Hippel–Lindau disease tumor suppressor; p53, cellular tumor antigen p53; p73, tumor protein p73; AICD, amyloid beta A4 protein; E2F1, transcription factor E2F1; HIF1α, hypoxia-inducible factor 3-alpha; BCA3, breast cancer-associated gene 3; RCAN1, regulator of calcineurin 1; EGFR, epidermal growth factor receptor; TβRII, transforming growth factor-beta receptor type II; PINK1, PTEN-induced putative kinase protein 1; CK1α, casein kinase 1 alpha; L11, ribosomal protein L11; S14, ribosomal protein S14; SHC, Src homology 2 domain-containing-transforming protein C1; HUR, Hu-antigen R; DrICE, *Drosophila* interleukin-1β-converting enzyme; Lag2, longevity-assurance protein 2; ML3, myeloid differentiation factor-2-related lipid-recognition domain protein 3.*

At the functional level, genetic experiments in *Schizosaccharomyces pombe* showed that the introduction of a specific *cullin1* mutant, which constitutively activates CRLs and therefore renders these CRLs independent from the neddylation machinery, was unable to rescue the phenotype of NEDD8 conjugation mutant *uba3-10* (Girdwood et al., 2011, 2012). This finding may suggest additional biological functions for neddylation that are impaired in *uba3-10* besides the neddylation defect of cullins.

### IDENTIFICATION OF NEDDYLATION SUBSTRATES - A DIFFICULT ISSUE

A major problem for the identification of new neddylation substrates is the high sequence similarity between NEDD8 and ubiquitin. Both proteins are sequence identical at their C-termini just downstream of a trypsin cleavage site resulting in an identical di-glycine footprint on a modified protein after trypsin digestion for proteomic analyses (**Figure 1**). Therefore, a di-glycine modification on a lysine of a given peptide cannot unanimously be attributed to either ubiquitin or NEDD8 conjugation.

Ubiquitin and NEDD8 also show a remarkable similarity in their three-dimensional structure and key residues are conserved between the two proteins, most prominently three amino acids (L8, I44, and V70) in the hydrophobic patch that are involved in mediating ubiquitin-protein interactions (Rao-Naik et al., 1998; Whitby et al., 1998; Choi et al., 2009; Girdwood et al., 2011). Thus, a high substrate specificity is required to avoid leakage of ubiquitin or NEDD8 into the respective other modification pathway. Indeed, it has become apparent in the last years that there is a crosstalk between the NEDD8 and ubiquitin conjugation machineries (Hjerpe et al., 2012b; Leidecker et al., 2012; Singh et al., 2012). NEDD8 can be activated by the ubiquitin E1 UBA1 and once activated is conjugated to substrates in a manner similar to ubiquitin (Singh et al., 2012). Inversely, however, NAE specifically activates NEDD8 and does not use ubiquitin as a substrate (Singh et al., 2012). NEDD8 is incorporated in the ubiquitin pathway by UBA1 when the ratio of free NEDD8 to free ubiquitin, which under normal conditions is close to one, shifts toward NEDD8 (Hjerpe et al., 2012a; Leidecker et al., 2012). The leakage of NEDD8 into the ubiquitin pathway leads to the formation of mixed chains with NEDD8 possibly functioning as chain terminator. This mechanism is likely the explanation for the identification of NEDD8 chains in a proteomic study by Jones et al. (2008) using overexpression of a tagged NEDD8 construct. The ability of NEDD8 to form chain linkages is not essential *in vivo* as demonstrated by the viability of a *Schizosaccharomyces pombe* strain carrying a Ned8p mutant construct where all lysine residues that could potentially engage in chain formation were mutated to alanine (Girdwood et al., 2011). Depletion of cellular ubiquitin levels can be caused by knockdown or inhibition of the 26S proteasome but can also have physiological causes such as temperature or oxidative stress (Hjerpe et al., 2012a; Leidecker et al., 2012). While this atypical neddylation has been proposed to act as a stress response to ubiquitin depletion, it is still unclear whether this type of atypical neddylation is biologically relevant *in vivo *(Hjerpe et al., 2012a; Leidecker et al., 2012).

### NOVEL NEDDYLATION SUBSTRATES

As outlined above, various studies have led to the identification of novel NEDD8-modified proteins. At the biochemical level, these proteins belong to different protein families including E3 ubiquitin ligases or transcription factors and neddylation has been shown to positively or negatively interfere with their activity (**Table 1**). Neddylation changes the biochemical properties of its target proteins by inducing conformational changes as well as allowing or precluding protein-protein interactions. Apart from its pleiotropic effects on protein degradation through CRL neddylation, neddylation has a prominent role in cell cycle regulation and cellular stress response pathways in human cells and has also been linked to Alzheimer and Parkinson disease (**Table 1**). In plants, the only known non-cullin neddylation substrates are DAMAGED DNA BINDING PROTEIN1 (DDB1) and ML3 from *Arabidopsis*. DDB1 is a subunit of a cullin4 E3 ubiquitin ligase and therefore biochemically close to the cells neddylation machinery (Hotton et al., 2012). ML3 is a protein with intriguing cell biological features that plays a role in pathogen responses (Hakenjos et al., 2013). Interestingly, there is also evidence that ML3 has the ability to bind neddylated proteins in a non-covalent manner. Since the precise biochemical function of ML3 remains to be determined, it is at present unclear what role neddylation has in the control of ML3 function.

Given the very recently acquired understanding of the close interplay between ubiquitylation and neddylation, new and old neddylation targets should undergo close scrutiny to ensure that they are genuine targets for NEDD8 modification (Hjerpe et al., 2012b). A catalog of appropriate characterization criteria has been published (Rabut and Peter, 2008).

## CONCLUSION

In this review, we have summarized the current knowledge of the neddylation pathway in eukaryotes with an emphasis on the role of neddylation in plants. While the enzyme pathway for the conjugation and deconjugation of NEDD8 has been elucidated in plants, findings from other eukaryotic model organisms suggest that there are more, unknown players in this pathway that need to be identified to gain a full understanding of the process and its regulation. Particularly the areas of NEDD8 processing, NEDD8 ligases and the identification of non-cullin NEDD8 substrates will require further detailed investigations in the future. Also the presence of ubiquitin-NEDD8 fusion proteins is unique to plants. The analysis of their processing could bear information that may allow understanding how the highly homologous ubiquitin and NEDD8 proteins were derived from each other during evolution.

## AUTHOR CONTRIBUTIONS

The authors have contributed in equal parts to the preparation of this manuscript.

## Conflict of Interest Statement

The authors declare that the research was conducted in the absence of any commercial or financial relationships that could be construed as a potential conflict of interest.
